# *Mycobacterium paragordonae* is an emerging pathogen in human pulmonary disease: clinical features, antimicrobial susceptibility testing and outcomes

**DOI:** 10.1080/22221751.2022.2103453

**Published:** 2022-08-08

**Authors:** Yuanchun Li, Wenping Zhang, Jing Zhao, Wenjie Lai, Yanlin Zhao, Yanming Li, Jiuxin Qu

**Affiliations:** a Department of Clinical Laboratory, National Clinical Research Center for Infectious Diseases, Guangdong Provincial Clinical Research Center for Infectious Diseases (Tuberculosis), Shenzhen Clinical Research Center for Tuberculosis, The Third People's Hospital of Shenzhen, Southern University of Science and Technology, Shenzhen, People’s Republic of China; b Department of Medical Imaging, National Clinical Research Center for Infectious Diseases, Guangdong Provincial Clinical Research Center for Infectious Diseases (Tuberculosis), Shenzhen Clinical Research Center for Tuberculosis, The Third People's Hospital of Shenzhen, Southern University of Science and Technology, Shenzhen, People’s Republic of China; c National Tuberculosis Reference Laboratory, Chinese Center for Disease Control and Prevention, Beijing, People’s Republic of China; d Department of Pulmonary and Critical Care Medicine, National Center of Gerontology, Chinese Academy of Medical Sciences, Beijing Hospital, National Health Commission; Institute of Geriatric Medicine, Beijing, People’s Republic of China

**Keywords:** *Mycobacterium paragordonae*, pulmonary disease, clinical features, antimicrobial susceptibility testing, outcomes

## Abstract

Objectives: *Mycobacterium paragordonae* (MPG) is an emerging and less common type of Non-tuberculous mycobacteria (NTM) and we know little about its characteristics and prognosis, hence we constructed this retrospective cohort study. Methods: MPG was identified using MALD-TOF MS, multi-target combined gene sequencing and WGS. Clinical information was collected, antimicrobial susceptibility testing was measured using the SLOMYCO panel, and optimal growth temperature testing was measured using Lowenstein-Jensen medium. Results: Eight MPGs were isolated from 1730 NTMs (0.46%); the mean age of MPG pulmonary disease (MPG-PD) patients was 42.38 ± 9.92 years, 37.5% were male, and the average BMI was 18.4 ± 0.51 kg/m^2^. All patients had the symptoms of cough and sputum and CT images mainly presented in patchy or streaky shadows, MPG grew at 25°C, 30°C and 37°C, and the optimal growth temperature is 37°C. MPGs were sensitive to clarithromycin, rifabutin, amikacin, linezolid, moxifloxacin, cotrimoxazole and ciprofloxacin, two isolates were resistant to rifampicin. Two patients had follow up information, their imaging remained stable during the follow-up. Conclusions: MPG-PD is a rare NTM disease and is more likely to develop in middle-aged, female, and low BMI patients. The patients present with no specific features within the symptoms as well as the CT imaging. The optimal growth temperature of MPG is at 37°C, MPG-PD has excellent sensitivity to drugs recommended by CLSI and presents with a stable disease.

## Highlights

1. MPG-PD is a very rare NTM disease within clinical practice, with an isolation rate of 0.46%;
2. MPG-PD is more likely to develop in middle-aged, female, and low BMI patients;
3. The MPG-PD patients presented with no specific features within the symptoms as well as the CT imaging;
4. MPG has a wide range of growth temperatures with an optimal growth temperature of 37°C;
5. MPG has excellent susceptibility to drugs recommended by CLSI for *in vitro* antimicrobial susceptibility testing, and patients with MPG-PD present with a stable disease that has a favourable prognosis.

## Background

Non-tuberculous mycobacteria (NTM), with more than 190 subspecies, is a large group of mycobacteria except for *Mycobacterium tuberculosis* complex and *Mycobacterium leprae* [1]. Generally, most NTM are considered to be saprophytic non-human pathogenic bacteria consisting of less virulent opportunistic pathogens; only approximately forty species are pathogenic to humans, accounting for approximately 25% of all NTM species [2].

*Mycobacterium gordonae* (MG), a slow-growing pigmented mycobacteria, rarely causes disease in humans [3]. MG has been known to cause infections, especially in patients with underlying predispositions or who have an immunosuppressive disease/condition such as AIDS, steroid therapy, and cancer, or in patients undergoing peritoneal dialysis, transplant recipients, and in children [4–6].

*Mycobacterium paragordonae* (MPG) was first isolated from the lungs of a patient in Korea in 2014 [7]. As with MG, MPG is also a slow-growing, pigmented (orange) bacterium that is widely found in the environment, such as soil and water, and is often considered a contaminating bacterium that can be recovered from freshwater, pipelines, and laboratory faucets [8, 9]. The DNA–DNA affinity comparison between MPG and the MG ATCC 14470 T standard strain was only 46.52%, which met the definition of a new species [7]. The phylogenetic tree of the *16S rRNA* gene sequences showed that the MPG isolates was most closely related to the MG ATCC 14470 T strain, with up to a 99.0% gene match; however, the two also had many inconsistencies in physicochemical characteristics, such as the incubation temperature. According to the relevant literature the optimum growth temperature of MG is 37°C; however, MPG does not grow at 37°C [7]. In addition, the MG colonies are yellow and the MPG colonies are orange in colour [7]. Furthermore, the urokinase test of MG is negative while MPG is positive [7].

MPG, like MG, is widely prevalent in the environment and is also an opportunistic pathogen[7]. Although MPG is relatively less virulent compared to other NTM such as *Mycobacterium kansasii*, associated pathogenic case reports have been published, MPG can be isolated from lung, spinal infection foci, and ascites specimens [9–11].

Currently, the literature regarding MPG disease is extremely limited, and although MPG disease cases have been reported, the pathogenesis, imaging features, and prognosis of these patients are poorly characterized; thus, the disease progression characteristics still need to be more clearly understood. In this study, MPG was accidentally isolated from NTM isolates at 37°C, the optimal growth temperature for most NTM isolates, which is conflicted with previous literature reports [7, 12]. Therefore, we retrospectively collected clinical, imaging, and follow-up data related to MPG to better understand MPG pulmonary disease.

## Methods

### Isolates collection and recovery, clinical data collection

The study population consisted of patients with suspected NTM identified by GenoType Mycobacteria CM assay (HAIN Life Sciences, Germany) in The Third People's Hospital of Shenzhen, Guangdong Province, China, from 2015 to 2020. The isolates isolated from the specimens were collected and stored at −80°C. Ethical approval was obtained from the Research Ethics Committee of the Third People’s Hospital of Shenzhen (No. 2021-035-02). Clinical information was collected according to the patient's electronic medical record, including baseline characteristic data, laboratory testing results, HRCT scan imaging, and follow-up information. The stored isolates were then thawed, inoculated, and grown in Lowenstein-Jensen medium at 37°C.

### Identification

#### MALDI-TOF MS

Matrix-Assisted Laser Desorption/Ionization Time-of-Flight Mass Spectrometry (MALDI-TOF MS) was used to identify the recovered isolates. A small number of bacteria was scraped onto a sterile inoculation loop and placed in a 1.5 mL EP tube containing 300 μL of 75% ethanol. After vortexing to inactivate the bacteria, the solution was centrifuged at 12000rpm for 5 min. The supernatant was discarded and the cell pellet was collected. Zirconia beads and acetonitrile were added to break the cell wall. 70% formic acid was added, and the samples were vortexed for 5 s then centrifuged at 12000rpm for 2 min. 1 μL of the supernatant was added to the MALDI-TOF MS target plate, and dried at room temperature. Finally, the surface was immediately covered with 1 μL of matrix solution, and dried at room temperature. The mass spectra of the isolates were compared against the Bruker's standard strain database using Bruker flex Analysis version 3.3.75.0 software (Bruker Daltonics GmbH, Germany).

#### Multi-target gene sequencing

The DNA of the isolates was extracted via a CTAB method. *16S rRNA*, *rpoB*, *Hsp65* and *16S-23S rRNA ITS* were amplified using multi-targeted gene sequencing combined with multi-targeted PCR (supplementary Table 1). The PCR reaction system was configured as follows: 2× Taq MasterMix 25 µL, primers (10 pmol/L) 1 µL each, DNA template 5 µL, and ddH_2_O 18 µL. The above PCR mixture was then analyzed under the following reaction conditions: pre-denaturation at 94°C for 5 min, denaturation at 94°C for 1 min, annealing at 60°C for 1 min, extension at 72°C for 1 min, 2–4 steps of 35 cycles, and finally full extension at 72°C for 10 min. The amplification products were then sent out for sequencing. The synthesis of the primers, purification of the amplification products and sequencing were all performed by Tsingke Biotechnology Co., Ltd. The sequencing results were compared using blast to complete the isolate identification.

**Table 1.** Identification of *Mycobacterium paragordonae* by MALD-TOF MS and multi-target combined gene sequencing.

Case number	MALD-TOF	*16s RNA*	*ITS*	*rpob*	*hsp65*	WGS	Final identification
1	MPG	/	MPG	MPG	MPG	/	MPG
2	MPG	/	MPG	MPG	MPG	/	MPG
3	MPG	/	MPG	/	MPG	MPG	MPG
4	MPG	/	MPG	MPG	MPG	MPG	MPG
5	MPG	/	MPG	MPG	MPG	MPG	MPG
6	MPG	/	MPG	MPG	MPG	MPG	MPG
7	M. avium	/	MPG	MPG	MPG	MPG	MPG
8	MPG	/	MPG	MPG	MPG	MPG	MPG

/: Sequencing failure.

#### Whole genome sequencing

The whole genome sequencing was conducted in Tianjin Biochip Corporation. The completed map sequences of *Mycobacterium paragordonae* 49061 (NZ_CP025546.1) and *Mycobacterium gordonae* DSM 43247 (NC_013441.1) were obtained from the NCBI database for average nucleotide identity (ANI) alignment and to construct a phylogenetic tree with the genomes of the clinical isolates in this study. The genomes were assembled into contigs using SPAdes (https://github.com/ablab/spades). The pairwise ANI values were determined from using pyani (https://github.com/widdowquinn/pyani) and visualized using the complex heatmap R package, and the species were identified according to the position distance of the evolutionary tree.

### High-resolution CT semi-quantitative scoring

Thin-section CT scans were obtained with the patients in the supine position, and was performed at end inspiration. The machine (Toshiba Asteion; Toshiba, Tokyo, Japan) parameters were as follows: 1.15 mm section thickness, 3 mm gap, 1 or 2-second scanning time per section, 120 kV, and 200 mA. The images were photographed at lung (window width, 1,800 HU, window level,400 HU). Two experienced radiologists reviewed the thin-section CT images respectively and reached a decision in consensus. The extent of disease at thin-section CT was evaluated. Each lung was divided into three lung zones: upper (above the carina), middle (below the carina up to the inferior pulmonary vein), and lower (below the inferior pulmonary vein) zones. Each lung zone was assigned a score that was based on the following: score 0, 0% involvement; score 1, less than 25% involvement; score 2, 25% to less than 50% involvement; score 3, 50% to less than 75% involvement; and score 4, 75% or greater involvement. Summation of scores provided overall lung involvement (maximal CT score for both lungs was 24).

### Optimal growth temperature testing

The isolates were inoculated into deionized water using a sterile scraping ring, dispersed into clumps using a Sensititre® turbidimeter, transferred to 3 tubes of Lowenstein-Jensen medium with 50 µL of suspension, and incubated at 25°C, 30°C, and 37°Cin a dark incubator for 7 days in order to observe the colour production and growth of the isolates.

### Antimicrobial susceptibility testing

The Sensititre® SLOMYCO 96-well plate containing 13 pre-made lyophilized drugs was used for the antimicrobial susceptibility testing, and the name and MIC concentration ranges of each antibiotic are shown in supplementary Table 2, in regards to the CLSI M24 and M62 documents. Freshly grown NTM were added into deionized water, turbidity was adjusted to 0.5 McFarland using a Sensitititre® turbidimeter, 50 µL of the suspension was transferred to a tube containing 11 mL of Sensitititre® Mueller Hinton broth medium, and the cap was replaced using a disposable spiking tip. The isolate was inoculated onto the plate according to the AutoInoculator®/AIM® instructions, sealed, laminated, and incubated at 37°C for 7 days before reading the results.

**Table 2.** - Baseline characteristics of *Mycobacterium paragordonae* pulmonary disease patients.

Baseline characteristics		Number (%)
Age (mean)		42.38 ± 9.92
Sex (man)		3(37.5%)
BMI (mean)		18.4 ± 0.51
Reasons for seeking medical support	CT abnormalities	5(62.5%)
	Suspected TB symptoms	3(37.5%)
	Proactive Screening	1(12.5%)
History of TB		3(37.5%)
Symptom	Cough	7(87.5%)
	Sputum	7(87.5%)
	Blood-stained sputum	2(25%)
Comorbidities	Bronchitis	1(12.5%)
	Pulmonary maculoplasm	1(12.5%)
	Diabetes	1(12.5%)
	Gout	1(12.5%)
CT examination	Bilateral lung	3(37.5%)
	Patchy or streaky shadows	7(87.5%)
	Nodules	2(25%)
	Bronchiectasis	1(12.5%)
	Calcified lymph nodes	0
	Pleural thickening	1(12.5%)
	Pleural effusion	1(12.5%)
Bacteriological examination	Smear	3(37.5%)
	Culture	8
	TSPOT	0
	IFN-γ	1(12.5%)
	MPB64	0
	TB-DNA	1(12.5%)
	Gene-XPERT	1(12.5%)
Treatment	HRZE	3(37.5%)
	Other antibiotics	2(25%)
	Untreated	3(37.5%)

H: Isoniazid, R:Rifampin, Z: Pyrazinamide, E: Ethambutol.

## Results

### Isolates identification and isolation

**Figure 1.**
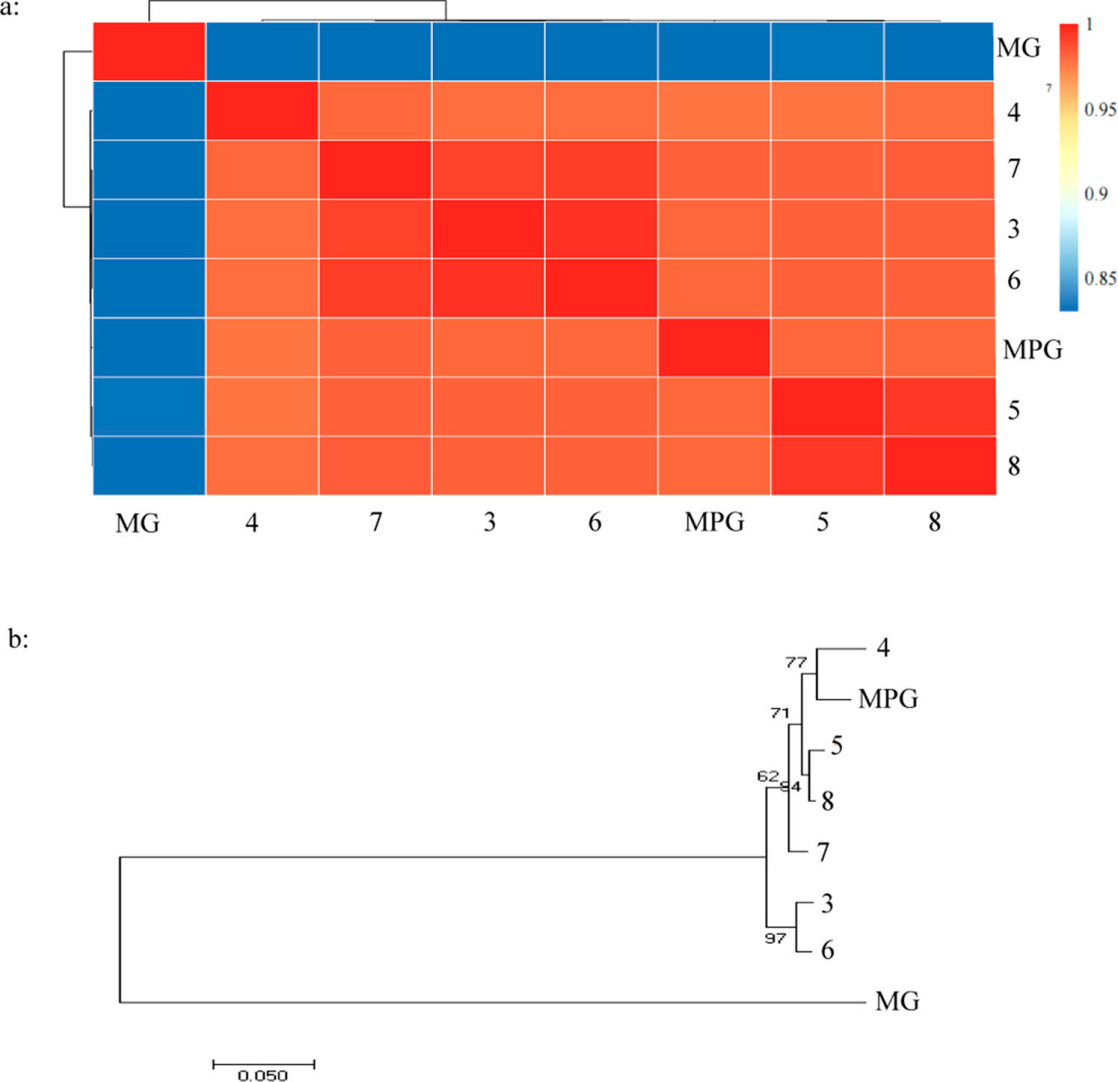
The identity of suspected *Mycobacterium paragordonae* clinical isolates by WGS. 1-A: Pairwise comparisons of ANIs of *Mycobacterium paragordonae* clinical isolates\ *Mycobacterium paragordonae* 49061 and *Mycobacterium gordonae*; 1-B: Phylogenetic tree of *Mycobacterium gordonae* and *Mycobacterium paragordonae* based on WGS constructed using the neighbor-joining method. MPG: *Mycobacterium paragordonae*, MG: *Mycobacterium gordonae* DSM 43247W.

A total of 1730 cases were initially identified as NTM by GenoType in The Third People's Hospital of Shenzhen from 2015 to 2020; among those 7 MPGs were identified by MALDI-TOF MS, as is shown in Table 1. The *16s RNA* sequencing was failed to classified the MPG from MG independently, while the whole 8 clinical isolates were identified to MG by multi-target genome (*ITS, hsp65* and *rpoB*) sequencing, which was further confirmed by whole genome sequencing, as is shown in Figure 1, the ANI values among suspected MPG isolates and MPG 49061 were all more than 95% while less than 90% among suspected MPG isolates and MG DSM 43247(Figure 1A), the same conclusion can also be drawn from evolutionary tree (Figure 1B), thus a total of 8 MPGs were finally included for further analysis, with an isolated rate of 0.46%.

### Baseline clinical characteristics

All MPG specimens isolated were of pulmonary origin, including 5 specimens from bronchoalveolar lavage fluid (62.5%) and 3 specimens from sputum (37.5%). As is shown in Table 2, patients with MPG were predominantly female, accounting for 62.5% of the total 5 cases with a mean age of 42.38 ± 9.92 years, and a mean body mass index (BMI) of 18.4 ± 0.51 kg/m^2^. The reasons for admission included 5 patients admitted for further examination due to abnormal CT scan images, 2 patients presented with blood-stained sputum and 1 patient presented with chest pain which were associated with suspected tuberculosis, and 1 patient was proactively admitted for early screening due to a history of exposure to tuberculosis patients. All patients have the symptoms with cough and sputum. Comorbidities included: hepatitis B in cases 2 and 6, diabetes mellitus in case 3, pulmonary maculopathy in case 4, and gout in case 6. Seven out of eight patients had HRCT scanning images and predominantly involved in unilateral lung lobes, with 87.5% of cases showing patchy or streaky shadows. The average HRCT semi-quantitative score was 8.80 (Table 3). Bacteriological findings were positive via sputum smear in only three cases, except for case 5 a patient having MPG combined with secondary TB. The remaining seven patients were negative for TB-related specific tests such as T-spot, MPB64, TB-DNA, and Gene-Xpert. Three of the eight patients were administered the HRZE standard anti-tuberculosis clinical regimen, while the remaining patients were not given the associated anti-mycobacterial treatment. Combining clinical manifestations, laboratory and imaging examinations, seven MPG-PD patients could be definitively diagnosed, and the additional patient could not be determined due to lack of information.

**Table 3.** HRCT semi-quantitative scores of the patients in this study.

Patients	Date	Upper		Middle		Lower		Total
		L	R	L	R	L	R	
103778	2017/12/18	0	1	0	0	1	1	3
106480	2018/6/21	2	1	1	1	1	2	8
104176	2018/4/25	2	2	1	1	1	1	8
104247	2020/6/14	0	4	1	2	0	1	8
106285	2018/7/2	0	4	1	1	0	1	7
103828	2018/1/31	3	4	1	2	0	0	10
	2018/9/23	3	4	0	2	0	0	9
	2019/5/19	3	4	0	1	0	0	8
	2020/1/6	3	4	0	1	0	0	8
	2021/5/10	3	4	0	1	0	0	8
107086	2018/12/17	0	4	1	1	0	0	6
	2019/9/9	0	4	1	1	0	0	6

L: left; R: right

### MPG optimal incubation temperature

**Figure 2.**
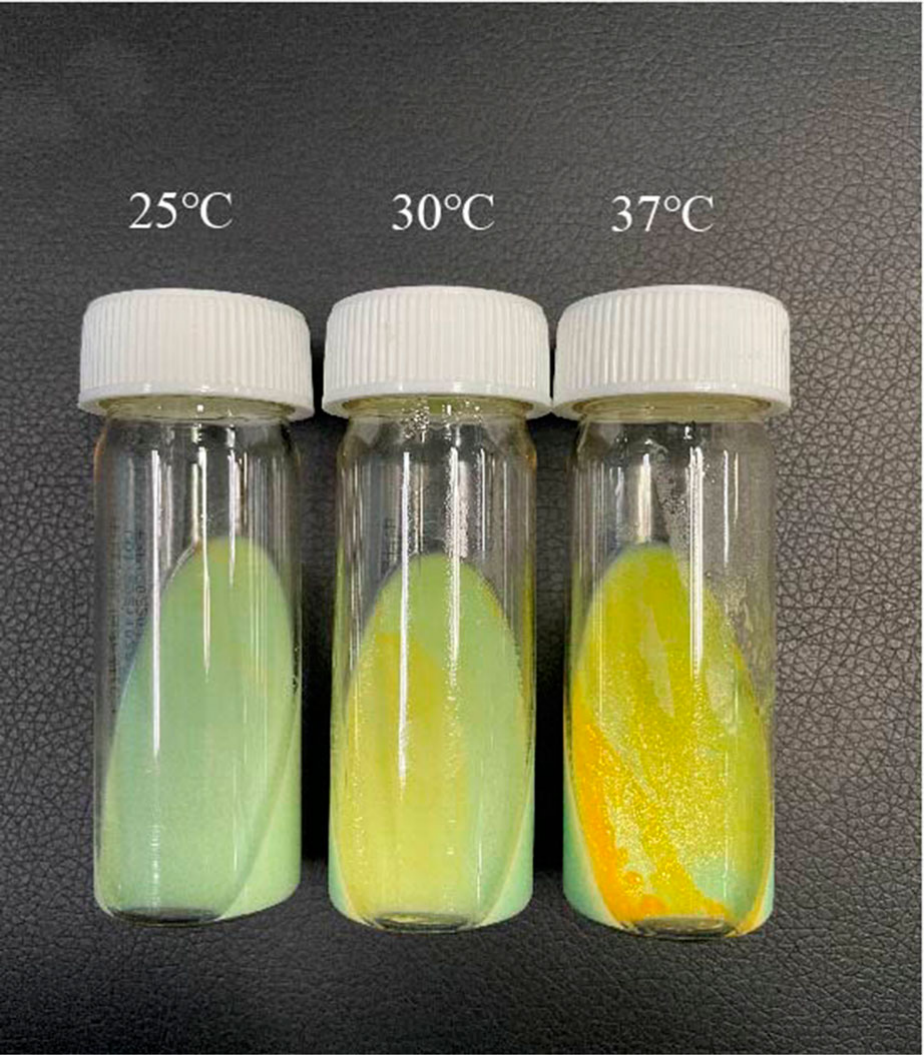
Growth of *Mycobacterium paragordonae* at different incubation temperatures.

As shown in Figure 2, MPG has minimal fine growth (almost invisible to the naked eye) and pigment deposition at 25°C on a slant medium. There was visibly more growth at 30°C with yellow colonies that can be easily distinguished. At 37°C, MPG growth was the most robust, with the colonies being moist and growing in overlapping clusters, thus giving them an orange colour.

### Antimicrobial susceptibility testing

As shown in Table 4, among the drugs recommended by CLSI that have clear cutoff values for antibiotic resistance, MPG is sensitive to clarithromycin, rifabutin, amikacin, linezolid, moxifloxacin, cotrimoxazole, and ciprofloxacin. Only two isolates of MPG were resistant to rifampicin.

**Table 4.** *In vitro* drug susceptibility of *Mycobacterium paragordonae* to recommended therapeutic agents.

Antibiotics	Drug resistance		
	S	I	R
Clarithromycin	8	0	0
Rifampin	6	/	2
Rifabutin	8	/	0
Amikacin	8	0	0
Linezolid	8	0	0
Moxifloxacin	8	0	0
Trimethoprim/sulfamethoxazole	8	/	0
Ciprofloxacin	8	0	0

### Short-term follow-up results of MPG pulmonary disease

**Figure 3.**
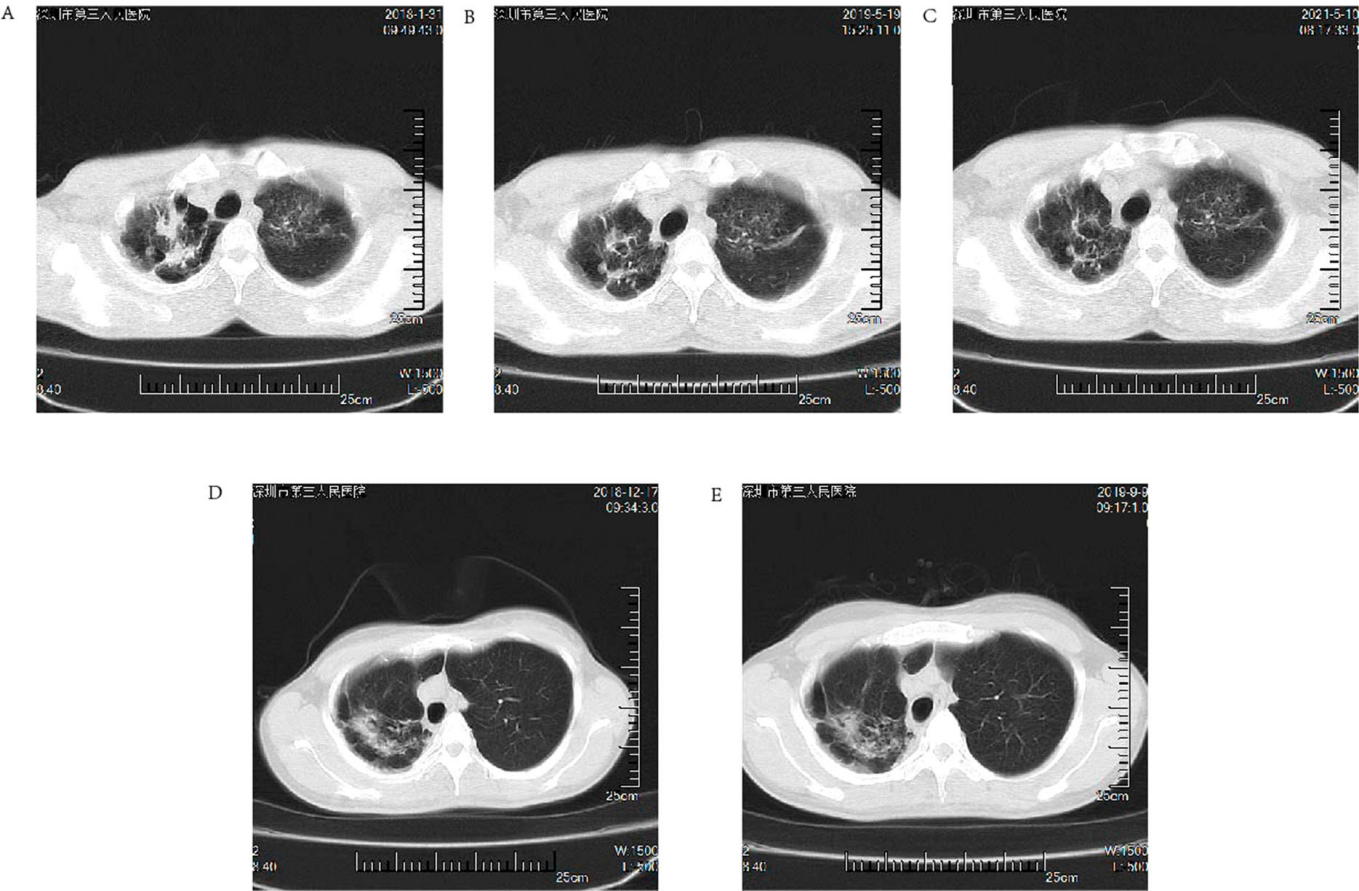
(A through E): Short-term follow-up HRCT images of patients with *Mycobacterium paragordonae* pulmonary disease; (A): HRCT images at the initial diagnosis in case 6; (B) HRCT images after the end of anti-tuberculosis treatment in case 6; (C) HRCT images 2 years after cessation of treatment in case 6; (D, E) HRCT images at the initial diagnosis and after 9 months of follow-up in case 8, respectively.

Two cases with follow-up information were tracked (case 6 and case 8) and both cases had a history of TB. Laboratory tests in case 6 were positive for TB-DNA, however, sputum culture was suggestive of an MPG infection, which was considered a case of MPG and TB co-infection; therefore, a regimen of rifampin, isoniazid, pyrazinamide, and ethambutol was given to case 6 for a total of 22 months, followed by 24 months of follow-up. After 22 months of antituberculosis treatment (Figure 3B) the lung lesions were absorbed and the imaging improved compared to the initial visit (Figure 3A), but 2 years after stopping treatment (Figure 3C) their lung lesions were approximately the same as when they first stopped therapy (Figure 3B).

Case 8 was in good health and had no laboratory evidence of tuberculosis disease, therefore no intervention was made and the patient was instructed to follow up regularly for a total of 9 months. Compared to the initial diagnosis (Figure 3D), the latest imaging results in case 8 were slightly more progressive than the previous results (Figure 3E). The patient’s overall situation was stable, and no interventions were made until the latest follow-up.

## Discussion

MPG and MP are closely related, and a phylogenetic tree based on *16S rRNA* gene sequences of Mycobacterium species shows a 99.0% similarity between the two species [7]. The sequence polymorphisms between MPG and MG ATCC 14470 T involved two nucleotides within the hypervariable region A, and three nucleotides in the hypervariable region B, respectively [7]. However, the DNA–DNA hybridization (DDH) values between MPG and MG ATCC 14470 T strain were 46.52 ± 0.7%, and according to the definition a DDH over 70% is considered as a different species [13], in our study, the ANI values either between MG DSM 43247W and MPG or between MPG clinical isolates and MG DSM 43247W were all less than 95%, therefore, MPG and MG are two different species with close affinity [7].

Phenotypic and molecular identification are the two main types of strain identification for NTM [14]. Traditional phenotypic identification can only distinguish NTM from TB, but not the exact strain of NTM; therefore, the identification of closely related NTM species can only be done on a molecular level.

MPG was first isolated from a Korean patient with pulmonary disease and identified using WGS[7]. In addition to the *16S rRNA* mentioned above, the similarity between MPG and the MG ATCC 14470 T strain using *hsp65* and *rpoB* sequences was also high at 95.9% and 95.1%, respectively[7]. Three combined gene sequences (*16S rRNA* +* hsp65* +*rpoB*) in tandem can distinguish MPG from MG. The MALDI-TOF MS profiles of MPG and MG ATCC 14470 T were also similar, and their presence or absence of small peak clusters at ∼m/z 2729 can identify MPG and MG[7]. The identification method used in this study, i.e. MALDI-TOF MS primary screening, and *16S rRNA* + *rpoB+ ITS + hsp65* sequencing was accurately identified, the identification capability was then further confirmed by WGS.

We searched and reviewed all the literatures on MPG culturing and found that the optimal growth temperature of MPG was controversial (Table 5). Kim showed that MPG grew well at 25°C but not at 37°C when MPG was first reported in 2014 [7]. In a series of subsequent MPG studies the culture conditions for MPG have all been performed at 30°C [12, 15, 16]. However, studies conducted by Kaelin *et al*. [8], Takajo *et al*. [9], and Moradi *et al*. [17] showed that MPG could also be isolated at an incubation temperature of 37°C. Furthermore, Azadi's study [18] showed that the optimum incubation temperature for MPG was 35°C. In our study, MPGs were isolated from NTM via a BD BACTEC MGIT 960 System at 37°C. Integrating the culture temperatures of the above studies and the experimental conditions in the existing laboratory, we incubated MPG at 25°C, 30°C, and 37°C for 7 days (Figure 1). Unlike the literature findings of the first report on MPG, MPG grew best and had the most intense pigment deposition at 37°C, suggesting that MPG is an NTM strain with low temperature requirements for growth. MPG can grow from 25°C∼37°C, meeting the growth conditions necessary for MPG to cause disease in humans. which is contrary to the findings of related literature [12]. This suggests to us that MPG has the ability to infect deeper tissues of the body, not just superficial tissues such as skin, and more attention should be paid.

**Table 5.** Literature review of *Mycobacterium paragordonae* incubation temperature.

References	Culture conditions (Growth)				Culture medium
	25°C	30°C	35°C	37°C	
Kim2014 [7]	√	\	\	×	7H10 agar slants
Kim2017 [12]	\	√	\	×	7H9 broth;7H10 agar plate
Kim2021 [15]	\	√	\	\	Middlebrook
7H10 agar media					
Kaelin2020 [8]	\	\	\	√	BD BACTEC MGIT 960,7H11 agar plates
Lee2020 [21]	\	√	\	\	7H10 agar plates
Takajo2020 [9]	\	\	\	√	BD BACTEC MGIT 960
Azadi2016 [18]	NA	NA	\	√	Lowenstein-Jensen (LJ)media
Moradi2019 [17]	√	\	\	√	Lowenstein-Jensen (LJ)media
Pereira2019 [10]	\	√	\	\	Middlebrook 7H10 medium
Cheung 2017 [20]	\	\	\	√	BD BACTEC MGIT 960
Kim2019 [12]	\	√	\	\	7H9 broth medium
Azadi2017 [19]	NA	NA	√	\	Lowenstein-Jensen (LJ)media

√: Grow, ×: Not grow, NA: Not available, \: Not measured.

Like MP, MPG is most commonly found in nosocomial environment, such as tap water, aerators/rectifiers connected to the faucet, endoscope-cleaning and disinfecting devices, and water storage tanks, which has been reported in Iran [18, 19], the United States [10], Switzerland [8], and so on. However, MPG is pathogenic to humans. In addition to the first Korean patient with MPG-PD, MPG was isolated from cerebrospinal fluid [11], sputum [9], stool [9], colonoscopy specimens [9] and infected ascites specimens [20].in our study, MPG were all from lung species, and there is close phylo-geographical context between the MPG from case 4 and MPG 49061, similarly present in the MPG isolated from case 3 and case 6, and the MPG isolated from case 5 and case 8. The absence of overlap in the timing of admission in these patients with close phylo-geographical context may exclude the possibility of laboratory contamination to some extent and may perhaps suggest a history of epidemiological exposure in these cases, further epidemiological investigation needed.

MPG clinical isolates showed excellent susceptibility to drugs used in treating common NTM diseases, and their clinical responsiveness has some effectiveness. In this study, among the drugs recommended by CLSI for *in vitro* antimicrobial susceptibility testing, only two isolates of MPG were resistant to rifampicin (25%), and all isolates were sensitive to clarithromycin, the first-line and most important antibiotic for NTM disease. In a report by Cheung *et al*. [20], a patient on peritoneal dialysis infected with MPG that was resistant to rifampicin and ciprofloxacin in an *in vitro* antimicrobial susceptibility testing was successfully treated with a modified regimen of azithromycin + amikacin + ethambutol based on the results of a antimicrobial susceptibility testing. The patient responded well with a change from cloudy to clear ascites.

MPG pulmonary infection has a favourable prognosis. In our study, the outcome of MPG patients is favourable even without using any MPG-specific therapeutic agents, as demonstrated by the lack of further worsening of the patients’ symptoms and HRCT imaging. The course of MPG lung disease is relatively stable, and we can propose treatment recommendations for less pathogenic NTM diseases such as MG disease based on the 2007 NTM disease treatment guidelines that include: several repeated positive cultures over several months, along with strong clinical and radiological evidence of disease being required to determine whether it needs immediate treatment. *In vitro* antimicrobial susceptibility testing is necessary before starting treatment. Although few susceptibility data is available, antimicrobial agents most consistently active *in vitro* include ethambutol, rifabutin, clarithromycin, linezolid, and fluoroquinolones [14]. An “individualized” assessment is vital when determining whether an MPG patient should be treated or not.

MPG also has value in other areas. In general, *Mycobacterium smegmatis* and *Mycobacterium indicus pranii* are NTM strains commonly used as candidates for live attenuated tuberculosis vaccines. MPG may have potential as a new anti-tuberculosis live attenuated vaccine against *Mycobacterium tuberculosis* and *Mycobacterium abscessus* due to its low virulence and wide temperature adaptation, prolonging the life span of infected dendritic cells, and thus facilitating antigen presentation [12]. In addition, Kim also tried to use MPG as a vehicle to develop new vaccines with new COVID-19 neutralizing antibodies for use against emerging virulent infectious diseases [15].

This study has the following limitations: first, the sample size is too small and may not be that representative, and the study is a relatively primary study with a simple study design, further studies are needed to reveal the characteristics and features of MPG-PD, moreover, LJ medium was used to evaluate the biological characteristics of MPG, similar to TB, MPG are also intracellular bacteria, and further study needed to explore whether their biological characteristics inside macrophages are consistent with those *in vitro*. Overall, our article briefly introduces MPG in a clinical context in the hope that clinicians will have a better understanding of MPG and NTM pulmonary diseases.

## Conclusion

MPG-PD is a very rare NTM disease in clinical practice. MPG-PD is may more likely to develop in middle-aged, female, and low BMI patients with no specific features within the symptoms or the CT images. MPG has a wide range of growth temperatures with optimal growth at 37°C. MPG has excellent sensitivity to antibiotics recommended by CLSI for *in vitro* antimicrobial susceptibility testing. Furthermore, patients with MPG-PD present with a stable disease that has a favourable prognosis.

## Author contributions

Yuanchun Li designed and conducted the research, analyzed the data and drafted the manuscript. Jiuxin Qu and Yanming Li are faculty advisors who helped with the research design, data analysis and manuscript. Wenping Zhang and Wenjie Lai conducted the research, Jing Zhao helped collect imaging information and performed data screening and analyzed, and Yanlin Zhao helped conduct the research. All the authors contributed to the article and approved the submitted version.

## Ethical approval

This study was approved by the Ethical Review Board of The Third People's Hospital of Shenzhen (2021-035-02).

## Supplementary Material

Supplemental Material
