# Supplementary material for: Mycobacterium paragordonae is an emerging pathogen in human pulmonary disease: clinical features, antimicrobial susceptibility testing and outcomes
Source: Emerg Microbes Infect. 2022 Aug 8;11(1):1973–81. doi: 10.1080/22221751.2022.2103453 (PMC9364734; doi:10.1080/22221751.2022.2103453)
Supplement: Supplemental Material [file TEMI_A_2103453_SM8776.docx]

**supplementary materials**

Table S1 Primers for multi-target gene sequencing

| Primer | Primer sequences (5’- 3’) | Primer fragment length (bp) |
| --- | --- | --- |
| *16S rRNA*-F | AGAGTTTGATCMTGGCTCAG | 1536 |
| *16S rRNA*-R | CCGTCAATTCMTTTRAGTTT |  |
| *rpoB*-F | GGCAAGGTCACCCCGAAGGG | 764 |
| *rpoB*-R | AGCGGCTGCTGGGTGATCATC |  |
| *ITS*-F | AAGTCGTAACAAGGTARCCG | ≈380 |
| *ITS*-R | TCGCCAAGGCATCCACC |  |
| *hsp65*-F | ATCGCCAAGGAGATCGAGCT | 644 |
| *hsp65*-R | AAGGTGCCGCGGATCTTGTT |  |

Table S2 MIC ranges of different drugs and their critical concentrations

| Antibiotics | MIC ranges(ug/ml) | Critical concentrations(ug/ml) |
| --- | --- | --- |
| Clarithromycin | 0.06-64 | 32 |
| Ethambutol | 0.5-16 | 8 |
| Rifabutin | 0.25-8 | 4 |
| Rifampicin | 0.12-8 | 8 |
| Streptomycin | 0.5-64 | 64 |
| Amikacin | 1-64 | 64 |
| Linezolid | 1-64 | 32 |
| Moxifloxacin | 0.12-8 | 4 |
| Doxycycline | 2-16 | / |
| Ciprofloxacin | 0.12-32 | / |
| Isoniazid | 0.25-8 | / |
| Etionamida | 0.3-20 | / |
| Trimethoprim-sulfamethoxazole | 0.12/238-8/152 | / |

MIC: minimum inhibitory concentration
